# A simple nomogram for predicting in-hospital mortality in patients with sepsis-induced coagulopathy in the ICU: a multicenter retrospective study

**DOI:** 10.3389/fmed.2026.1884945

**Published:** 2026-10-29

**Authors:** Ruimin Tan, Yuan Yuan, Quansheng Du, Jingmei Wang, Donglin Fu, Shujia Zhang

**Affiliations:** 1 Department of Critical Care, Chongqing Academy of Medical Sciences, Chongqing General Hospital, Chongqing University, Chongqing, China; 2 Department of Emergency Medicine and Critical Care Medicine, Hebei General Hospital, Shijiazhuang, Hebei, China; 3 Critical Care Department, Handan Central Hospital, Handan, Hebei, China

**Keywords:** ICU, in-hospital mortality, multicenter retrospective study, nomogram, sepsis-induced coagulopathy

## Abstract

**Background:**

One typical consequence of sepsis is sepsis-induced coagulopathy (SIC), which is linked to a significantly higher risk of in-hospital death for patients in the intensive care unit (ICU). The specificity, sensitivity, and usability of current clinical prognostic evaluation techniques are restricted, and nomogram-based models are still not widely used to predict outcomes in patients with SIC. In order to increase the accuracy of forecasting in-hospital mortality in ICU patients with SIC, this study developed and validated a straightforward and user-friendly nomogram based on multicenter retrospective data.

**Methods:**

480 SIC patients who were admitted to the ICUs of Hebei General Hospital and Handan Central Hospital (East District) between March 1, 2021, and March 1, 2024, were retrospectively recruited and randomly assigned to a training set and a testing set at a 7:3 ratio. An external validation set consisted of an additional 229 SIC patients who were hospitalized to Chongqing General Hospital's intensive care unit between March 1, 2021, and January 1, 2026. Eight kinds of variables were gathered, such as laboratory values, comorbidities, and demographics. Eight kinds of information were gathered, including laboratory measurements, comorbidities, and demographics. Using least absolute shrinkage and selection operator (LASSO) regression, core predictors were first chosen. Multivariable logistic regression analysis was then used to find independent risk factors linked to SIC patients’ in-hospital mortality. Using R's rms package, a nomogram was created based on the final set of chosen predictors. Receiver operating characteristic (ROC) curves, calibration plots, and decision curve analysis (DCA) were used to assess the model's performance in terms of discrimination, calibration, and clinical utility.

**Results:**

Among the 480 SIC patients, the total in-hospital death rate was 42.7%. Five independent predictors—including coronary artery disease, serum potassium levels, prothrombin time–international normalized ratio (PT-INR), platelet count, and platelet transfusion—were identified as being associated with in-hospital mortality among patients with SIC. With area under the ROC curve (AUC) values of 0.873 [95% Confidence Interval (CI): 0.832–0.914] in the training set, 0.776 (95% CI: 0.696–0.856) in the testing set, and 0.756 (95% CI: 0.692–0.819) in the validation set, the nomogram showed high discriminative capacity. DCA demonstrated that the model offered a high net therapeutic benefit over a broad range of threshold probabilities (0.1–1.0), and calibration plots revealed good agreement between projected and observed probability.

**Conclusions:**

The nomogram created in this work has strong discrimination, good calibration, and acceptable clinical applicability, making it possible to estimate in-hospital mortality risk in ICU patients with SIC in an understandable and accurate manner. By helping doctors identify high-risk patients early on and provide tailored care plans, this tool may maximize the use of medical resources and possibly lower mortality rates among SIC patients.

## 1 Introduction

Disrupted immune modulation and organ damage are the main characteristics of sepsis, a potentially fatal disease marked by widespread organ failure brought on by a dysregulated host response to infection (1). A severe imbalance between the coagulation system and systemic inflammatory reactions is the hallmark of sepsis-induced coagulopathy (SIC), a frequent consequence of sepsis (2). The idea of SIC was formally presented by the International Society on Thrombosis and Haemostasis (ISTH) in 2017 and integrated into the sepsis diagnostic framework (3). According to epidemiological data, between 24% and 60% of sepsis patients globally experience SIC; in China, this frequency is significantly greater, at 67.9% (4). SIC is more likely to occur in patients in the intensive care unit (ICU) who are older, have underlying comorbidities, or have more severe illnesses, such as raised acute physiology and chronic health evaluation II (APACHE II) scores (5). SIC can develop into disseminated intravascular coagulation (DIC) if it is not treated quickly and properly. This can result in a significant rise in in-hospital mortality, with reported fatality rates almost doubling (6).

Endothelial damage, the cascade release of inflammatory mediators, and excessive coagulation factor activation are just a few of the interrelated events that make up the intricate pathophysiology of SIC (7). There is a two-way amplification loop between coagulation and inflammation: increased inflammatory reactions quicken the coagulation cascade, and coagulation activation worsens inflammation even more (8). The risk of death can be significantly increased by this vicious cycle, which can lead to extensive microvascular thrombosis, microcirculatory failure, and a bleeding tendency before developing into DIC and multiple organ dysfunction syndrome (MODS).

Currently, standard laboratory indicators and traditional physiological grading systems are the main tools used in clinical practice to assess the prognosis of patients with SIC. Nevertheless, these instruments have poor sensitivity, specificity, and usefulness for practical clinical use (9). Even though the SIC score, sepsis-associated coagulopathy score, and a number of coagulation-related biomarkers and genetic indicators have shown promise for risk stratification and outcome prediction, the majority of current research is limited to single-center sets or particular populations, which results in poor model generalizability and limited sample representativeness (10, 11).

A nomogram is a graphical tool that allows for the customized prediction of clinical event probability without the requirement for computing support by converting intricate mathematical models and biological or clinical data into an understandable visual format (12). Its use in the early risk assessment of SIC has been investigated in a number of recent studies, which have shown a respectable degree of accuracy in predicting the risk of in-hospital SIC development in sepsis patients. However, there is still a dearth of data specifically pertaining to ICU populations (13).

In light of this, the current study uses multicenter retrospective data to create a straightforward and clinically useful nomogram-based prediction tool. This model aims to increase the accuracy of in-hospital mortality prediction among patients with SIC by using easily accessible clinical characteristics and pertinent biomarkers. Clinical usability is given special attention, with the aim of supporting quick risk assessment and personalized early warning to offer doctors intuitive decision support, enabling prompt intervention and therapeutic strategy improvement. This study aims to improve the predictive framework's robustness and generalizability by overcoming the drawbacks of earlier models based on single-source data and limited sample sizes through the aggregation and analysis of multicenter data.

## 2 Materials and methods

### 2.1 Data source

Patients with SIC who were admitted to the ICUs of Hebei General Hospital and Handan Central Hospital (East District) between March 1, 2021, and March 1, 2024, and for whom reasonably full clinical data were available, were included in this retrospective investigation. Based on their in-hospital results upon discharge, patients were divided into two groups: those who died and those who survived. At a ratio of 7:3, the dataset was split at random into training and testing sets. Additionally, SIC patients with comparatively complete clinical data who were admitted to Chongqing General Hospital's ICU between March 1, 2021, and January 1, 2026, were retroactively included as an external validation set for independent model validation. The Ethics Committees of Hebei General Hospital (No. 2026-LW-076), Handan Central Hospital (No. 2026057), and Chongqing General Hospital (No. KY S2024-037-01) approved the ethical criteria that were followed in conducting this study. Every study process adhered to pertinent institutional ethical guidelines and the Declaration of Helsinki's tenets.

### 2.2 Study population

Patients with SIC diagnoses from three tertiary healthcare facilities were included in this multicenter retrospective analysis. First admission to the ICU, a diagnosis of sepsis based on the Sepsis-3 criteria (defined as life-threatening organ dysfunction resulting from a dysregulated host response to infection), and a diagnosis of SIC based on the ISTH's 2017 definition were all prerequisites for eligibility. According to the ISTH 2017 criteria, SIC was diagnosed based on platelet count, prothrombin time–international normalized ratio (PT-INR), and Sequential Organ Failure Assessment (SOFA) score, with a total SIC score ≥4 indicating the presence of SIC. Patients who were younger than 18 years old, had an ICU stay of less than 24 h or passed away within 24 h of ICU admission, had pre-existing coagulation disorders or thrombocytopenic conditions (such as immune thrombocytopenic purpura or hemophilia), or showed signs of coagulation abnormalities prior to ICU admission, such as pregnancy, hematologic malignancies, or post-cardiopulmonary resuscitation status were all excluded. Additionally, cases with unclear ICU outcomes, insufficient clinical or laboratory data, or rejection of active therapy were not included. Figure 1 shows the comprehensive patient enrolling procedure.

**Figure 1:**
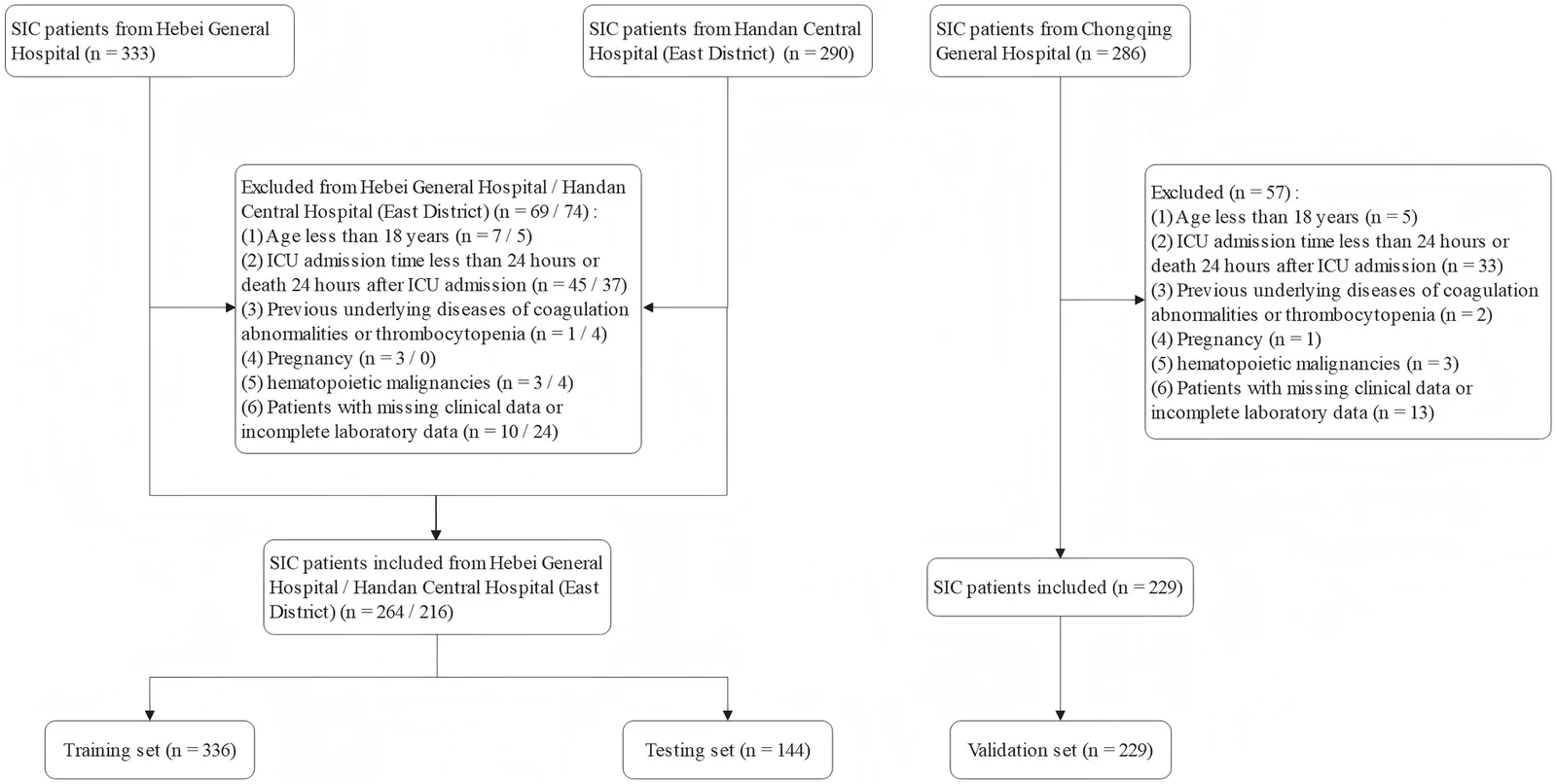
Flow diagram of patient selection and cohort construction. Patients with SIC admitted to the participating ICUs were screened according to the predefined inclusion and exclusion criteria. Eligible patients from Hebei General Hospital and Handan Central Hospital were included in the development cohort and randomly divided into the training and testing sets at a 7:3 ratio. Eligible patients from Chongqing General Hospital constituted the independent external validation cohort. SIC, sepsis-induced coagulopathy; ICU, intensive care unit.

### 2.3 Data extraction

Clinical information was taken out of the three participating medical facilities’ electronic medical record systems. Variables were collected across eight predefined domains: demographic characteristics (sex and age); baseline comorbidities, including coronary artery disease, hypertension, diabetes mellitus, chronic obstructive pulmonary disease, cerebrovascular disease, hepatic dysfunction, chronic kidney disease, malignancy, and recent surgical history; primary sites of infection (respiratory tract, abdominal cavity, bloodstream, urinary tract, central nervous system, skin and soft tissue, and other locations); commonly used ICU severity scores, including the APACHE II score and the SOFA score; physiological parameters, including body temperature, heart rate, blood pressure indices (systolic, diastolic, and mean arterial pressure), and respiratory rate; laboratory measurements, including arterial blood gas parameters, hepatic and renal function tests, procalcitonin (PCT) levels, a comprehensive coagulation profile (e.g., prothrombin time [PT], activated partial thromboplastin time [APTT], fibrinogen, and thrombin time), complete blood count parameters, and serum electrolyte levels (potassium, sodium, chloride, calcium, phosphorus, and magnesium); clinical interventions, including central venous access, mechanical ventilation, anticoagulant therapy, corticosteroid administration, use of vasoactive agents, continuous renal replacement therapy, and transfusion of blood products; and hospitalization-related variables, including total length of hospital stay and length of ICU stay.

According to the 2017 ISTH criteria, T0 was defined as the time of the initial diagnosis of SIC. For each patient, laboratory measurements, physiological parameters, and clinical intervention-related variables recorded within 24 h after T0 were retrieved, and the first available measurement or recorded intervention status during this period was used as the baseline value. Platelet transfusion was defined as platelet administration occurring within the first 24 h after SIC diagnosis and was considered an early treatment-related variable reflecting the severity of coagulation dysfunction. Thus, biochemical variables were defined at the individual-patient level rather than using group-level mean values or optimal cutoff values. When multiple measurements were available within the 24-hour window, only the first recorded value was used to ensure a consistent baseline assessment across patients. Accordingly, the present model was designed as an early prognostic model among patients with confirmed SIC based on information available during the initial 24-hour period after SIC diagnosis and did not incorporate longitudinal trajectories of coagulation parameters or time-dependent responses to subsequent therapies during the ICU stay.

### 2.4 Statistical analysis

The Shapiro–Wilk test was used to determine whether continuous variables were normal. The independent-samples t test was used to compare data between groups that followed a normal distribution, which is shown as the mean ± standard deviation. The Wilcoxon rank-sum test was used to compare non-normally distributed data, which are reported as the median with interquartile range. The chi-square test was used to compare categorical variables, which are reported as counts and percentages.

Candidate predictors were prespecified from routinely available and clinically relevant domains, including demographic characteristics, comorbidities, physiological parameters, laboratory measurements, disease severity scores, and clinical interventions. A total of 73 candidate predictors were initially considered. All candidate variables were then simultaneously entered into a least absolute shrinkage and selection operator (LASSO) regression model. The L1 regularization penalty shrinks weakly informative coefficients toward zero and was used to reduce variable redundancy and the influence of correlations among candidate predictors, thereby improving model parsimony and stability. The optimal penalty parameter (*λ*) was determined by 10-fold cross-validation using the minimum cross-validation error criterion. Predictor selection was primarily data-driven; clinical relevance was considered when defining the initial candidate variable set and when interpreting the final model, rather than being used to manually force individual variables into or out of the model.

Multicollinearity among the seven variables retained by LASSO regression was assessed using variance inflation factors (VIFs), and no substantial collinearity was observed. Seven variables retained by LASSO regression were subsequently entered into a multivariable logistic regression model with an entry criterion of 0.05 and a removal criterion of 0.10 to identify the final predictors of in-hospital mortality. The final multivariable logistic regression model included five predictors and was supported by 138 in-hospital mortality events in the training cohort, corresponding to an events-per-variable ratio of 27.6. A nomogram was then constructed using the rms package. The point scale for each predictor was derived from its regression coefficient and its contribution to the linear predictor of the final logistic regression model. The individual predictor scores were summed to obtain the total points, which were subsequently mapped to the predicted probability of in-hospital mortality. To evaluate the discriminative performance of the model, receiver operating characteristic (ROC) curves were created, and the area under the curve (AUC) as well as the corresponding sensitivity and specificity at the ideal cutoff value were computed. Plotting calibration curves using the rms package and running the Hosmer–Lemeshow goodness-of-fit test were two other ways to assess calibration. The model's performance in the original dataset is represented by the apparent line, the performance estimated from 1,000 bootstrap resamples is represented by the bias-corrected line, and perfect agreement between predicted and observed outcomes is indicated by the diagonal “ideal” line in the calibration plot. In addition, bootstrap stability analysis was performed to evaluate the robustness of the variable selection procedure. In each bootstrap sample, the complete modeling process, including LASSO-based variable selection and subsequent multivariable logistic regression analysis, was repeated. The selection frequency of each predictor included in the final nomogram was calculated. Using the rmda program, decision curve analysis (DCA) was carried out to measure the model's net clinical benefit over a variety of threshold probabilities. The “None” line in the DCA plot indicates the approach of assuming that no patients will experience the event, resulting in a net benefit of zero, while the “All” line reflects the strategy of assuming that all patients will experience the event, resulting in a negative slope of net benefit. When a model's curve is above both reference lines over a significant range of threshold probabilities, it is deemed clinically helpful. In order to evaluate the nomogram's prediction performance and generalizability, it was lastly externally evaluated in a separate validation set. Calibration performance was additionally quantified using calibration-in-the-large, calibration slope, and Brier score with 95% confidence intervals in the training cohort, testing cohort, and independent Chongqing external validation cohort. Continuous variables were entered into the models using their original clinical scales without additional transformation or standardization.

R software (version 4.3.1) and SPSS software (version 27.0) were used for all statistical analyses. Statistical significance was defined as a two-sided *P* value of less than 0.05.

### 2.5 Design of the validation set

An external validation set was created utilizing a retrospective sample of SIC patients admitted to Chongqing General Hospital's ICU between March 1, 2021, and January 1, 2026 in order to evaluate the applicability and stability of the suggested model. This set was subjected to the same inclusion and exclusion criteria as previously mentioned. 229 patients were the intended final sample size for the validation set. Figure 1 depicts the comprehensive patient selection procedure.

## 3 Results

### 3.1 Baseline characteristics

Following screening, 480 SIC patients were enrolled in the trial; 205 of them died in the hospital, resulting in a 42.7% overall mortality rate. Patients were randomly randomized at a 7:3 ratio to a training set and a testing set using R software (version 4.3.1). There were 138 non-survivors (41.07%) among the 336 patients in the training group and 67 non-survivors (46.53%) among the 144 patients in the testing set. Supplementary Table S1 shows the two sets’ baseline characteristics. Patients in the training set had lower levels of serum phosphorus and greater levels of alanine aminotransferase, salt, and chloride when compared to the testing set; these changes were statistically significant (*P* < 0.05). For the remaining clinical variables, there were no statistically significant variations between the two groups (*P* > 0.05), suggesting that the training and testing sets were generally comparable and representative.

The baseline clinical features of the 336 SIC patients in the training set are compiled in Table 1. Hepatic dysfunction, cancer, and concomitant coronary artery disease were more common in non-survivors (*n* = 138) than in survivors (*n* = 198). Lactate, total bilirubin, direct bilirubin, alanine aminotransferase, aspartate aminotransferase, serum potassium, and blood urea nitrogen were all considerably higher in non-survivors. Higher rates of platelet, red blood cell, and cryoprecipitate transfusion were also noted, as were prolonged prothrombin time and activated partial thromboplastin time, as well as increased PT-INR and red cell distribution width. Non-survivors, on the other hand, had reduced rates of plasma and human albumin transfusion as well as lower levels of total protein, albumin, monocytes, hemoglobin, platelet count, and plateletcrit. The survivor and non-survivor groups did not vary substantially in any other characteristics (*P* > 0.05).

**Table 1:** Baseline comparison of death group and survival group in training set.

Variables	Death group (*n* = 138)	Survival group (*n* = 198)	Z/*χ*^2^	*P*
Demographic data				
Male, n(%)	84 (60.87)	124 (62.63)	χ^2^ = 0.11	0.744
Age, years, M (Q_1_, Q_3_)	72.00 (61.00, 82.00)	73.00 (63.00, 80.75)	Z = −0.30	0.763
Underlying diseases, n(%)				
Coronary heart disease	37 (26.81)	30 (15.15)	χ^2^ = 6.93	0.008
Hypertension	61 (44.20)	84 (42.42)	χ^2^ = 0.10	0.746
Diabetes	34 (24.64)	46 (23.23)	χ^2^ = 0.09	0.766
Chronic obstructive pulmonary disease	18 (13.04)	21 (10.61)	χ^2^ = 0.47	0.493
Cerebrovascular disease	48 (34.78)	61 (30.81)	χ^2^ = 0.59	0.444
Liver dysfunction	75 (54.35)	71 (35.86)	χ^2^ = 11.31	<0.001
Chronic kidney disease	40 (28.99)	48 (24.24)	χ^2^ = 0.95	0.331
Malignancy	21 (15.22)	15 (7.58)	χ^2^ = 4.96	0.026
History of surgery within 3 months	58 (42.03)	100 (50.51)	χ^2^ = 2.35	0.126
Infection sites, n(%)				
Pulmonary	99 (71.74)	125 (63.13)	χ^2^ = 2.71	0.100
Abdominal	52 (37.68)	91 (45.96)	χ^2^ = 2.28	0.131
Blood	24 (17.39)	24 (12.12)	χ^2^ = 1.84	0.174
Urinary system	23 (16.67)	27 (13.64)	χ^2^ = 0.59	0.443
Central nervous system	3 (2.17)	3 (1.52)	χ^2^ = 0.00	0.976
Skin and soft tissue	2 (1.45)	8 (4.04)	χ^2^ = 1.10	0.294
Other	7 (5.07)	9 (4.55)	χ^2^ = 0.05	0.823
ICU disease severity scores, M (Q_1_, Q_3_)				
APACHE score	23.00 (17.00, 27.75)	23.00 (18.00, 28.00)	Z = −0.20	0.843
SOFA score	9.00 (7.00, 12.00)	9.00 (7.00, 11.00)	Z = −1.43	0.154
Vital signs, M (Q_1_, Q_3_)				
Temperature(℃)	36.50 (36.00, 37.48)	36.50 (36.00, 37.08)	Z = −0.60	0.550
HR(times/min)	101.50 (85.25, 118.75)	101.00 (84.25, 114.75)	Z = −0.66	0.511
SBP(mmHg)	120.00 (103.50, 140.00)	122.00 (105.25, 138.75)	Z = −0.45	0.654
DBP(mmHg)	66.00 (56.00, 78.00)	67.00 (59.00, 74.75)	Z = −0.25	0.801
MAP(mmHg)	85.00 (75.33, 95.25)	85.50 (75.00, 95.33)	Z = −0.25	0.801
RR(times/min)	21.00 (16.25, 28.00)	20.00 (15.00, 26.00)	Z = −0.99	0.324
Laboratory tests, M (Q_1_, Q_3_)				
pH	7.37 (7.27, 7.43)	7.36 (7.30, 7.41)	Z = −0.42	0.675
lactate(mmol/L)	3.29 (2.02, 5.20)	2.40 (1.62, 4.24)	Z = −3.39	<0.001
AB(mmol/L)	20.50 (17.00, 24.28)	20.15 (16.83, 23.87)	Z = −0.31	0.759
OI	201.41 (133.50, 282.33)	215.07 (163.12, 292.10)	Z = −1.33	0.183
TP(g/L)	−4.69 (−8.15, −1.12)	−4.85 (−7.77, −1.54)	Z = −0.08	0.938
ALB(g/L)	48.35 (42.82, 52.32)	50.15 (44.23, 56.70)	Z = −2.19	0.028
TBIL(*μ*mol/L)	26.65 (23.02, 29.37)	27.85 (23.38, 32.40)	Z = −2.55	0.011
DBIL(μmol/L)	26.50 (16.57, 50.27)	18.75 (12.20, 29.85)	Z = −3.79	<0.001
ALT(U/L)	47.00 (23.00, 105.50)	25.00 (15.48, 62.80)	Z = −3.77	<0.001
AST(U/L)	90.50 (37.50, 185.50)	49.00 (29.15, 99.03)	Z = −4.17	<0.001
K(mmol/L)	4.10 (3.61, 4.50)	3.83 (3.50, 4.20)	Z = −2.92	0.004
Na(mmol/L)	142.00 (136.00, 147.00)	141.00 (136.35, 144.15)	Z = −1.54	0.123
Cl(mmol/L)	106.00 (102.00, 112.00)	106.80 (103.00, 111.00)	Z = −0.35	0.725
Ca(mmol/L)	1.96 (1.85, 2.12)	1.96 (1.83, 2.13)	Z = −0.59	0.558
PO_4_(mmol/L)	1.13 (0.79, 1.59)	1.03 (0.74, 1.40)	Z = −1.41	0.159
Mg(mmol/L)	0.82 (0.72, 0.92)	0.81 (0.69, 0.92)	Z = −1.07	0.286
BUN(mmol/L)	14.71 (9.22, 21.83)	11.95 (7.97, 19.13)	Z = −2.29	0.022
Cr(μmol/L)	127.60 (85.30, 189.75)	125.25 (76.45, 198.05)	Z = −0.45	0.651
PCT(ng/mL)	19.70 (3.00, 61.81)	15.38 (3.23, 51.85)	Z = −0.63	0.526
PT(s)	16.90 (14.60, 21.78)	16.00 (14.10, 17.50)	Z = −3.51	<0.001
PT-INR	1.48 (1.29, 1.84)	1.45 (1.31, 1.55)	Z = −2.07	0.038
APTT(s)	40.45 (33.65, 47.15)	36.45 (32.70, 43.60)	Z = −2.34	0.019
FIB(g/L)	5.08 (3.08, 13.33)	5.04 (3.33, 10.93)	Z = −0.07	0.942
TT(s)	15.55 (4.44, 17.78)	15.85 (6.92, 16.88)	Z = −0.29	0.773
WBC(10^9^/L)	11.21 (5.60, 17.52)	11.66 (6.34, 18.08)	Z = −0.20	0.839
NEU(10^9^/L)	9.91 (4.80, 16.13)	10.28 (5.19, 16.75)	Z = −0.18	0.861
LYM(10^9^/L)	0.49 (0.28, 0.78)	0.50 (0.34, 0.81)	Z = −0.87	0.382
MON(10^9^/L)	0.29 (0.11, 0.48)	0.36 (0.17, 0.54)	Z = −2.06	0.040
Hb(g/L)	98.00 (83.25, 120.00)	109.50 (90.25, 128.75)	Z = −3.41	<0.001
RDW(fL)	49.65 (46.12, 56.08)	47.85 (45.00, 53.92)	Z = −2.26	0.024
PLT(10^9^/L)	48.00 (36.00, 93.75)	104.50 (90.00, 133.00)	Z = −8.74	<0.001
PDW	16.35 (13.22, 17.50)	15.50 (13.20, 17.10)	Z = −1.41	0.158
Plateletcrit	0.07 (0.04, 0.12)	0.12 (0.10, 0.15)	Z = −7.92	<0.001
P-LCR	11.80 (10.60, 12.80)	11.55 (10.80, 12.60)	Z = −0.70	0.483
MPV(fL)	38.80 (30.80, 45.88)	37.10 (31.00, 44.20)	Z = −0.97	0.331
Interventions, n(%)				
Deep venous catheterization	113 (81.88)	164 (82.83)	χ^2^ = 0.05	0.823
Anticoagulant drugs	55 (39.86)	92 (46.46)	χ^2^ = 1.44	0.230
Mechanical ventilation	122 (88.41)	173 (87.37)	χ^2^ = 0.08	0.776
Hormones	103 (74.64)	144 (72.73)	χ^2^ = 0.15	0.696
Vasoactive drugs	134 (97.10)	187 (94.44)	χ^2^ = 1.35	0.246
CRRT	86 (62.32)	102 (51.52)	χ^2^ = 3.85	0.050
Platelet transfusion	58 (42.03)	30 (15.15)	χ^2^ = 30.39	<0.001
Red blood cell transfusion	78 (56.52)	76 (38.38)	χ^2^ = 10.78	0.001
Plasma infusion	87 (63.04)	98 (49.49)	χ^2^ = 6.03	0.014
Infusion of cold precipitated clotting factors	27 (19.57)	20 (10.10)	χ^2^ = 6.05	0.014
Human Fibrinogen Infusion	112 (81.16)	140 (70.71)	χ^2^ = 4.74	0.029
Infusion of human albumin	4 (2.90)	0 (0.00)	χ^2^ = 3.61	0.058
Hospitalization Status				
ICU Stay(Days)	8.00 (3.25, 13.75)	5.00 (3.00, 12.75)	Z = −1.67	0.095
Length of hospital stay(Days)	16.00 (8.25, 28.00)	16.00 (9.00, 27.00)	Z = −0.69	0.491

Z: Mann–Whitney test, χ^2^: Chi-square test.

M: Median, Q_1_: 1st Quartile, Q_3_: 3st Quartile.

APACHE II: acute physiology and chronic health evaluation II; SOFA: sequential organ failure assessment; HR: heart rate; SBP: systolic blood pressure; DBP: diastolic blood pressure; MAP: mean arterial pressure; RR: respiratory rate; AB, actual bicarbonate; OI, oxygenation index; TP: total protein; ALB: albumin; TBIL: total bilirubin; DBIL: direct bilirubin; ALT: alanine aminotransferase; AST: aspartate aminotransferase; K: potassium; Na: sodium; Cl: chloride; Ca: calcium; PO_4_: phosphorus; Mg: magnesium; BUN: blood urea nitrogen; Cr: creatinine; PCT: procalcitonin; PT: prothrombin time; PT-INR: prothrombin time-international normalisation ratio; APTT: activated partial thromboplastin time; FIB: fibrinogen; TT: thrombin time, WBC: white blood cells; NEU: neutrophils; LYM: lymphocytes; MON: monocytes; Hb: hemoglobin; RDW: red blood cell distribution width; PLT: platelet; PDW: platelet distribution width; P-LCR: platelet large cell ratio; MPV: mean platelet volume; CRRT: continuous renal replacement therapy; ICU: intensive care unit.

### 3.2 Feature selection

LASSO regression was used in the training set to select variables and simplify the model. Using ten-fold cross-validation based on the smallest mean squared error, the ideal penalty parameter (*λ*) was found to minimize overfitting. Figure 2A depicts the dynamic changes in variable coefficients during the LASSO selection process, and Figure 2B shows how the best *λ* value is chosen during cross-validation. In the end, LASSO regression revealed seven factors that were most strongly linked to in-hospital mortality among SIC patients. These included three laboratory indicators (serum potassium, PT-INR, and platelet count); two clinical procedures (platelet transfusion and red blood cell transfusion); and two baseline comorbidities (hepatic dysfunction and coronary artery disease).

**Figure 2:**
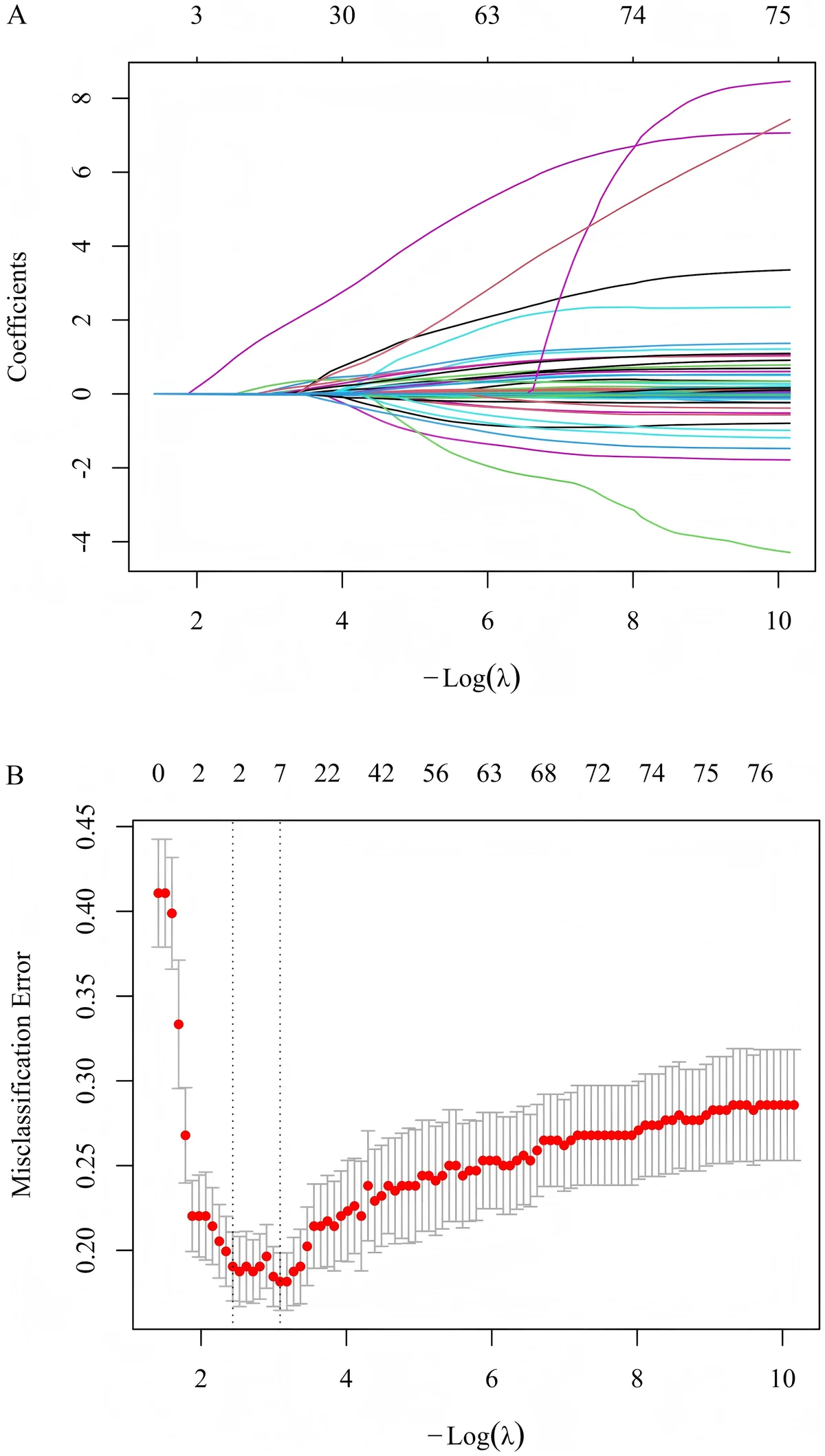
Predictor selection using LASSO regression in the training set. **(A)** LASSO coefficient profiles of the candidate predictors. Each curve represents the change in the coefficient of one candidate predictor as the regularization parameter (*λ*) varies. **(B)** Selection of the optimal *λ* using 10-fold cross-validation. The optimal penalty parameter was determined according to the minimum cross-validation error criterion and was subsequently used to identify predictors retained by the LASSO model. LASSO, least absolute shrinkage and selection operator.

To find factors independently linked to in-hospital mortality among patients with SIC, the variables chosen by LASSO regression were then stepwise entered into a multivariable logistic regression model with an entry criterion of 0.05 and a removal criterion of 0.10. The multivariable analysis identified five independent predictors: coronary artery disease [Odds Ratio (OR) = 2.119, 95% CI: 1.031–4.355, *P* = 0.041], serum potassium (OR = 1.881, 95% CI: 1.199–2.950, *P* = 0.006), PT-INR (OR = 50.386, 95% CI: 13.241–191.733, *P* < 0.001), platelet count (OR = 0.961, 95% CI: 0.952–0.970, *P* < 0.001), and platelet transfusion (OR = 2.095, 95% CI: 1.085–4.046, *P* = 0.028). For PT-INR, the reported OR corresponds to a 1.0-unit increase because the variable was modeled continuously on its original scale. Bootstrap stability analysis demonstrated that the five predictors retained in the final nomogram showed high selection frequencies across repeated resampling, including PT-INR (98.4%), platelet count (96.7%), platelet transfusion (91.2%), serum potassium (86.5%), and coronary artery disease (72.8%) (Supplementary Table S3). Detailed results are presented in Table 2.

**Table 2:** Multivariable logistic regression analysis of factors associated with in-hospital mortality in patients with SIC.

Variables	β	SE	Wald	*P*	OR(95% CI)
Coronary heart disease	0.751	0.367	4.179	0.041	2.119 (1.031–4.355)
Serum potassium	0.632	0.23	7.575	0.006	1.881 (1.199–2.950)
PT-INR	3.92	0.682	33.048	<0.001	50.386 (13.241–191.733)
PLT	−0.04	0.005	67.319	<0.001	0.961 (0.952–0.970)
Platelet transfusion	0.74	0.336	4.856	0.028	2.095 (1.085–4.046)
Constant	−5.747	1.37	17.599	<0.001	

β: Partial regression coefficient; SE: Standard Error; OR: Odds Ratio; CI: Confidence Interval.

SIC: sepsis-induced coagulopathy; PT-INR: prothrombin time-international normalisation ratio; PLT: platelet.

### 3.3 Nomogram construction

A nomogram (Figure 3) was constructed based on the five predictors retained in the final multivariable logistic regression model. The point scale assigned to each predictor was derived proportionally from its regression coefficient and its contribution to the linear predictor of the final model, rather than being determined subjectively. An individual score is obtained for each variable by projecting its observed value onto the corresponding point scale. The individual scores are then summed to obtain the total points, which are mapped to the probability scale to estimate the individual risk of in-hospital mortality. This graphical representation enables clinicians to obtain an individualized risk estimate using routinely available clinical information without performing the underlying regression calculation directly.

**Figure 3:**
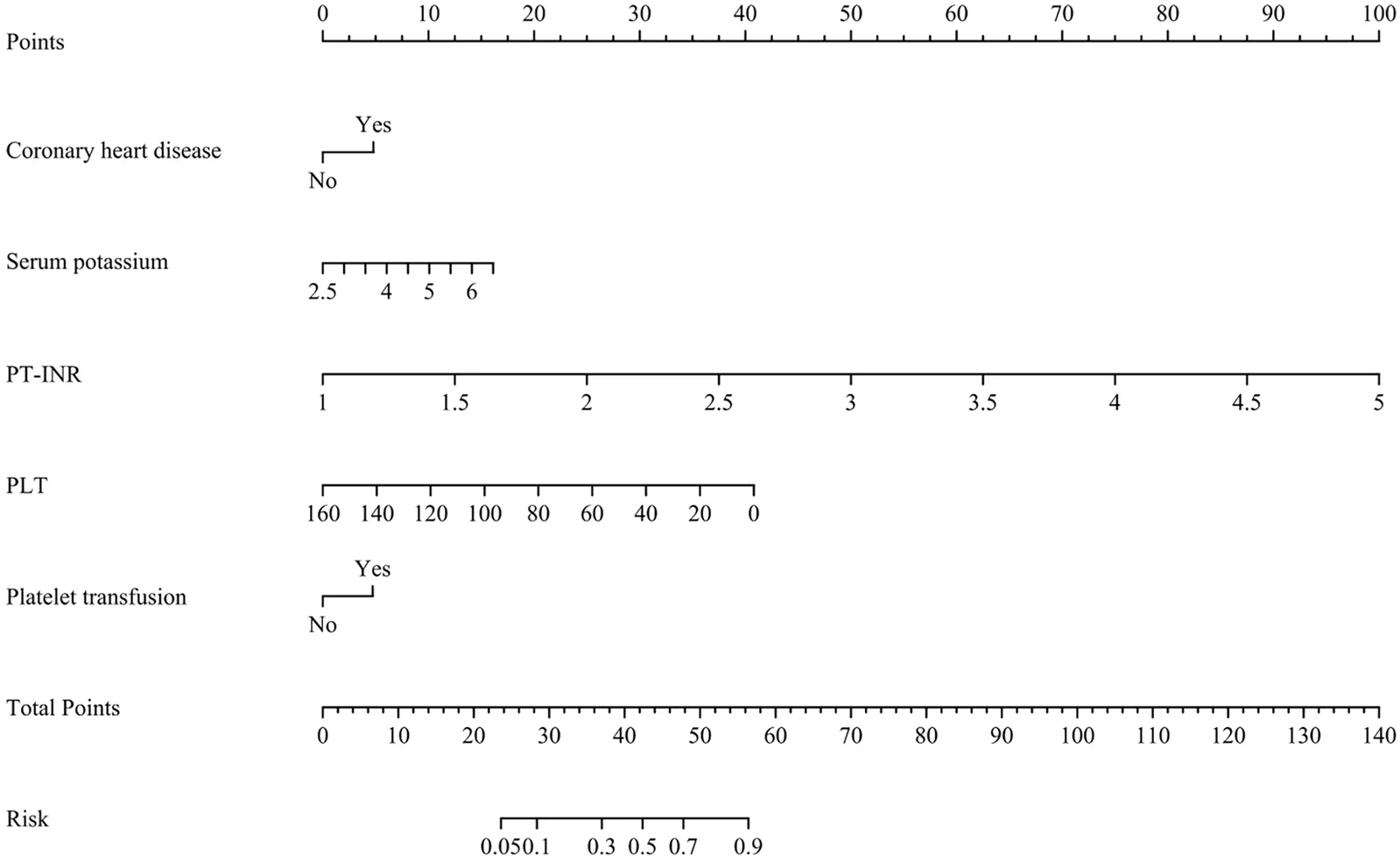
Nomogram for predicting in-hospital mortality in patients with sepsis-induced coagulopathy. The nomogram was constructed using the five predictors retained in the final multivariable logistic regression model: coronary heart disease, serum potassium, PT-INR, PLT, and platelet transfusion. For each patient, the value of each predictor is projected onto the corresponding point scale, and the individual points are summed to obtain the total score. The total score is then mapped to the predicted probability of in-hospital mortality. SIC, sepsis-induced coagulopathy; PT-INR, prothrombin time–international normalized ratio; PLT, platelet count.

### 3.4 Model performance validation

#### 3.4.1 Validation set

For the external validation set, 286 SIC patients were first discovered during the study period. 57 patients were eliminated from the analysis after the exclusion criteria were applied. These included 5 patients under the age of 18, 33 patients who had an ICU stay of less than 24 h or who passed away within 24 h of ICU admission, 2 patients with pre-existing coagulation disorders or thrombocytopenic conditions, 1 pregnant patient, 3 patients with hematologic malignancies, and 13 patients with incomplete clinical or laboratory data. In the end, 229 patients made up the validation set, and 102 of them died in the hospital, yielding a mortality rate of 44.54%. Supplementary Table S2 provides a summary of the baseline characteristics of the pertinent predictive factors in the validation set.

#### 3.4.2 ROC curves and AUC values

With an AUC of 0.873 (95% CI: 0.832–0.914), the nomogram showed good discriminative ability in the training set. The AUC was 0.776 (95% CI: 0.696–0.856) in the testing set and 0.756 (95% CI: 0.692–0.819) in the external validation set. In the external validation set, the sensitivity, specificity, positive predictive value, and negative predictive value were 0.696, 0.717, 0.664, and 0.746, respectively. These findings indicate that the model retained acceptable discriminative performance in the independent external validation cohort. No formal head-to-head comparison between the nomogram and individual biochemical markers was performed; therefore, these findings should not be interpreted as demonstrating statistical superiority of the nomogram over any single laboratory parameter. A visual depiction of model performance is provided by the ROC curves for the nomogram in the training, testing, and validation sets, which are displayed in Figures 4, S1A, and S1B, respectively. Table 3 summarizes specific metrics for each set, including AUC, 95% confidence intervals, optimal cutoff values, sensitivity, specificity, positive predictive value, and negative predictive value.

**Figure 4:**
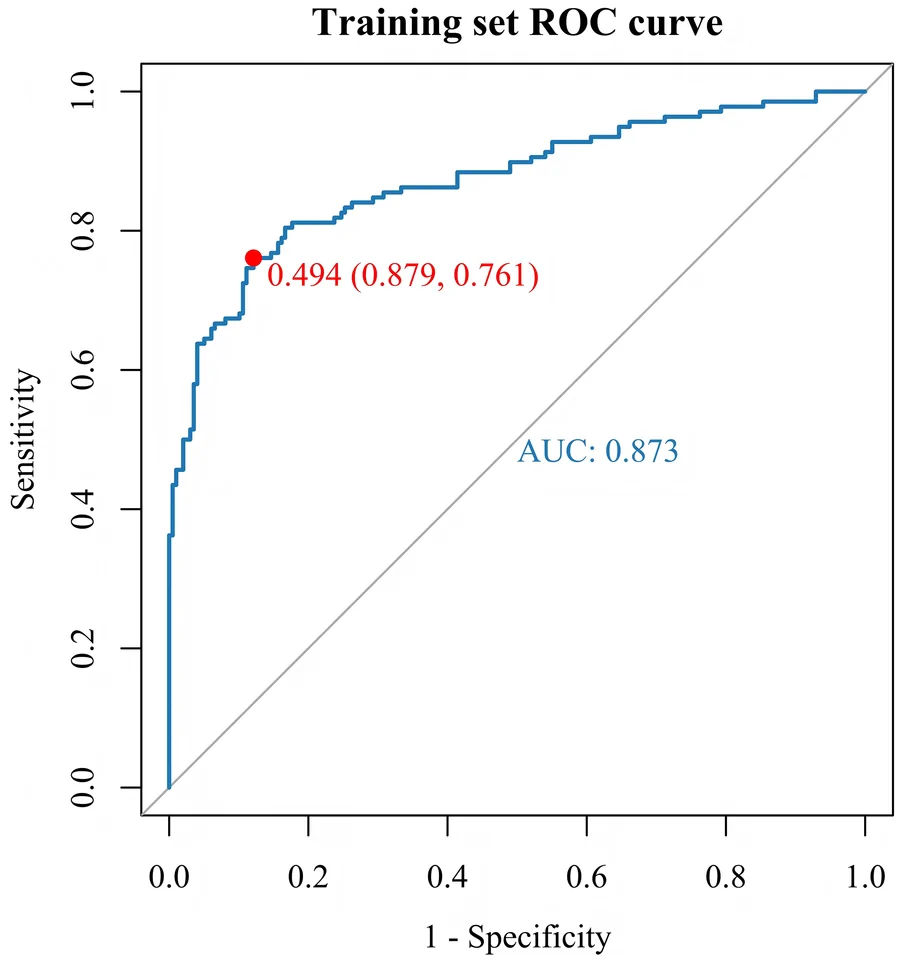
ROC curve of the nomogram for predicting in-hospital mortality in the training set. The nomogram achieved an AUC of 0.873 (95% CI: 0.832–0.914), indicating good discriminative performance in the training cohort. ROC, receiver operating characteristic; AUC, area under the curve; CI, confidence interval.

**Table 3:** Detailed results of the nomogram model for each dataset.

Dataset	AUC	95% CI	Optimal cutoff value	Sensitivity	Specificity	PPV	NPV
Training set	0.873	0.832–0.914	0.494	0.761	0.879	0.814	0.841
Testing set	0.776	0.696–0.856	1.093	0.552	0.935	0.881	0.706
Validation set	0.756	0.692–0.819	−0.698	0.696	0.717	0.664	0.746

AUC: area under the curve; CI: confidence interval; PPV: positive predictive value; NPV: negative predictive value.

#### 3.4.3 Model calibration

Good model reliability was indicated by calibration analysis, which showed a high degree of agreement between predicted and observed probabilities across all sets (Table 4). Figures 5, S2A, and S2B display the calibration plots for the training, testing, and validation sets, respectively. Quantitative calibration assessment further demonstrated that calibration-in-the-large values were close to zero, calibration slopes remained close to one, and Brier scores indicated acceptable prediction accuracy across the training, testing, and Chongqing external validation cohorts (Supplementary Table S4). These results imply that the nomogram offers precise risk assessment for SIC patients’ in-hospital mortality.

**Table 4:** Evaluation of the calibration of the nomogram model across datasets.

Dataset	Brier scores	Calibration errors
Training set	0.133	0.036
Testing set	0.197	0.066
Validation set	0.210	0.088

**Figure 5:**
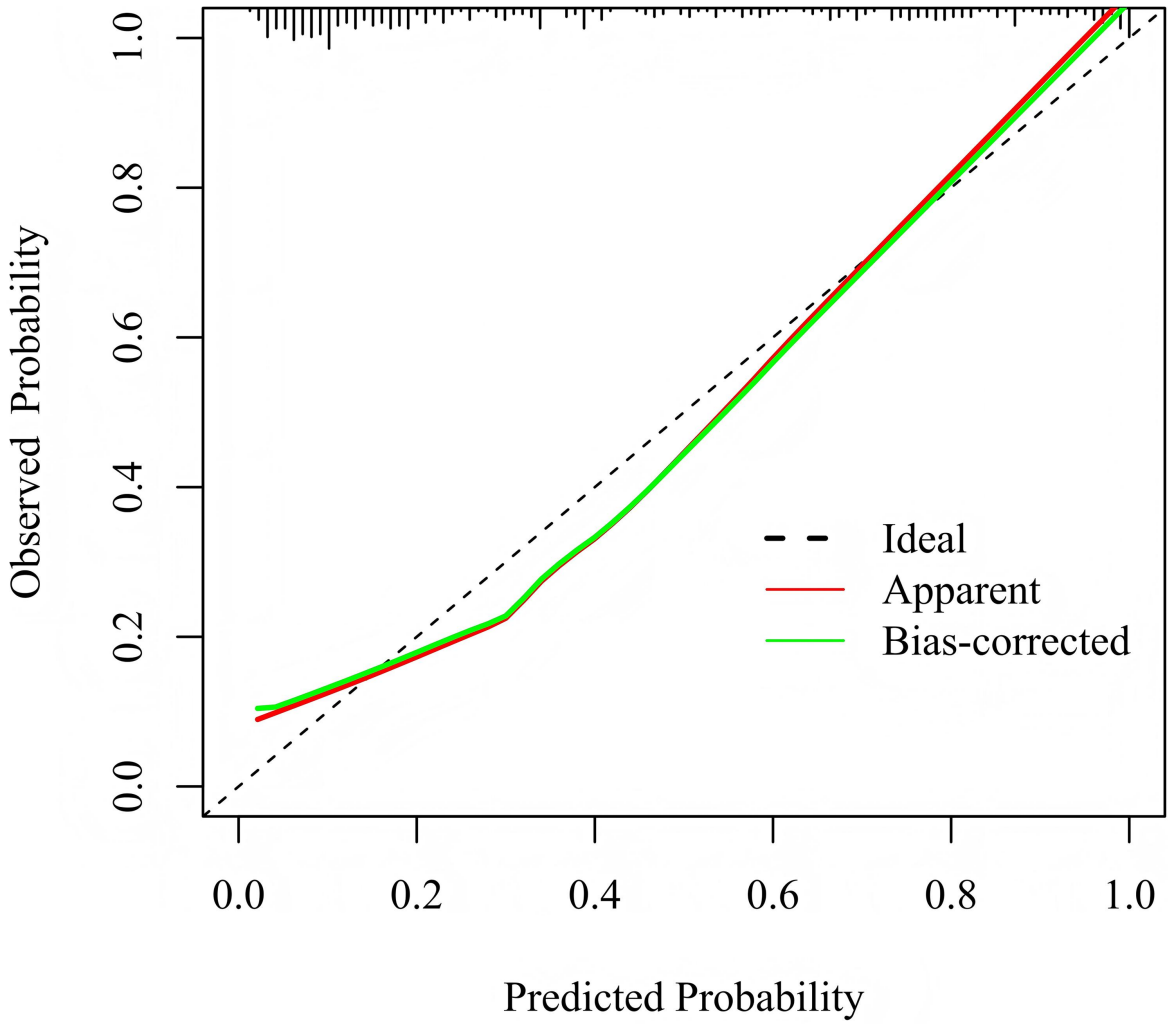
Calibration plot of the nomogram for predicting in-hospital mortality in the training set. The diagonal ideal line represents perfect agreement between predicted and observed probabilities. The apparent line represents the calibration performance of the model in the original training data, whereas the bias-corrected line represents the calibration estimate after 1,000 bootstrap resamples. Closer agreement between the model curves and the ideal line indicates better calibration.

#### 3.4.4 DCA curves

The prediction model in the training, testing, and external validation sets underwent DCA; the outcomes are displayed in Figures 6, S3A, and S3B, respectively. The threshold probability is represented by the *x*-axis and the net benefit by the *y*-axis in the DCA plots. The findings show that the nomogram offers a greater net benefit across the majority of threshold probability ranges, indicating strong clinical value for determining the risk of in-hospital mortality among SIC patients. Interestingly, the model curve falls substantially above the “None” and “All” reference lines within the threshold range of 0.1 to 1.0, suggesting that using the model within this range can help doctors make better intervention choices while reducing overtreatment or missed diagnoses. The model's resilience and generalizability across heterogeneous datasets were further demonstrated by the fact that it maintained a favorable net benefit in both the testing and validation sets.

**Figure 6:**
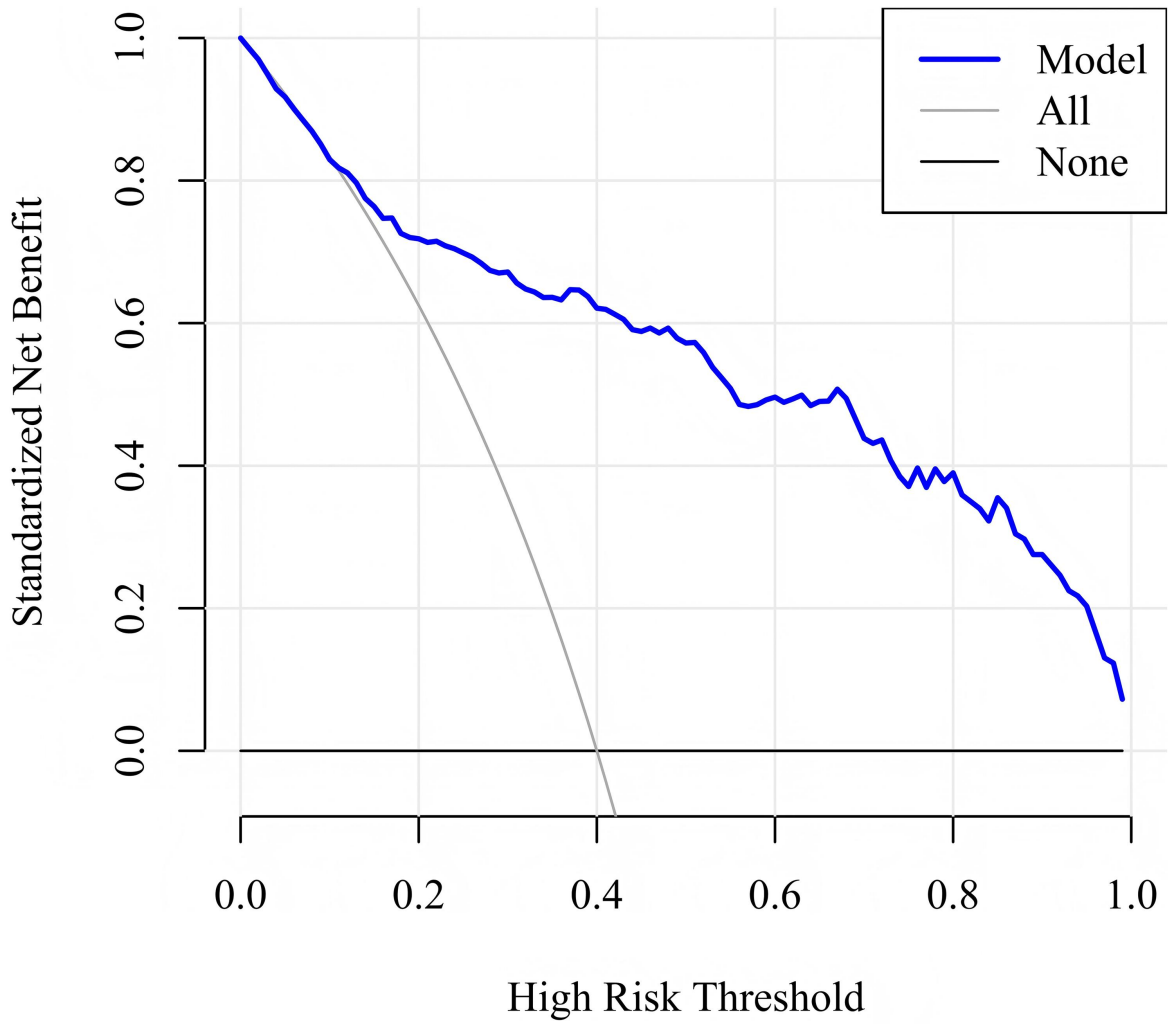
DCA of the nomogram for predicting in-hospital mortality in the training set. The *x*-axis represents the threshold probability and the *y*-axis represents net benefit. The nomogram curve shows the net benefit of applying the prediction model across different threshold probabilities. The “None” reference line represents the strategy of assuming that no patients will experience the outcome, whereas the “All” reference line represents the strategy of assuming that all patients will experience the outcome. A model curve above both reference strategies indicates potential clinical net benefit within the corresponding threshold range. DCA, decision curve analysis.

## 4 Discussion

In this study, we used multicenter real-world clinical data to create and externally evaluate a nomogram for predicting in-hospital mortality among ICU patients with SIC. In terms of clinical application, calibration, and discrimination, the model showed consistent performance. It demonstrated significant net benefit across a broad range of threshold probabilities in DCA and attained acceptable discrimination in the external validation set, suggesting its potential use as a complementary prognostic tool in clinical practice. Rather than replacing established severity assessment systems, this nomogram was developed to provide additional individualized mortality risk stratification among patients who had already fulfilled SIC criteria. This nomogram could be a useful tool to help clinicians identify high-risk patients early and optimize customized treatment plans.

SIC is thought to be a key pathogenic feature that connects organ dysfunction, coagulation dysregulation, and inflammatory reactions as sepsis progresses. SIC essentially represents a significant imbalance between the hemostatic system and the host immunological regulatory network during viral stress (14, 15). By activating monocytes/macrophages and neutrophils through pattern recognition receptors, persistent release of pathogen-associated molecular patterns and damage-associated molecular patterns can cause the overproduction of pro-inflammatory cytokines and tissue factor, which in turn drives aberrant amplification of the coagulation cascade (16). A persistent procoagulant condition is the consequence of endothelium damage impairing natural anticoagulant systems, including as the protein C system, antithrombin, and tissue factor pathway inhibitors (17). Widespread microvascular thrombosis formation impairs tissue perfusion and oxygen delivery, leading to cellular energy failure and mitochondrial malfunction, which in turn accelerates the development and progression of MODS (18). Clinically, the appearance of SIC frequently indicates that the patient has reached a high-risk stage, which is marked by a vicious cycle of self-amplifying “inflammation–coagulation–hypoxia.” This cycle significantly raises the risk of irreversible organ failure and in-hospital mortality among ICU patients in addition to hastening the accumulation of organ damage. Thus, SIC's significant involvement in prognostic models can be explained mechanistically by taking into account it as a major mediator of in-hospital mortality risk.

Serum biomarkers and clinical grading systems are crucial instruments for determining the prognosis and severity of the disease in the clinical management of patients with SIC, although both have significant drawbacks. While lactate levels more directly indicate impaired microcirculatory perfusion and cellular hypoxia, which are closely correlated with short-term mortality risk, PCT is widely considered an indirect marker of systemic infection and bacterial load, with its dynamic changes reflecting responsiveness to antimicrobial therapy (19, 20). Nevertheless, the pathophysiological process of SIC, which is essentially characterized by coagulation–anticoagulation imbalance, is only partially covered by these biomarkers, which mostly capture discrete features of inflammation or metabolic condition. Additionally, PCT and lactate are less stable for risk categorization due to their susceptibility to temporary effects from clinical measures like fluid resuscitation or source control. More significantly, a single biomarker lacks a thorough evaluation of organ functional reserve for predicting in-hospital mortality, making it impossible to separate the causative relationship between inflammation-driven coagulation anomalies and primary organ harm.

The SOFA and SIC scores have been extensively utilized in prognostic studies, and multidimensional clinical scoring systems provide a standardized framework for SIC patient evaluation. The SOFA score shows strong mortality discrimination at the population level and quantitatively assesses several organ function domains, capturing both the severity of the disease and its dynamic progression (21). By combining platelet count, coagulation parameters, and organ function indices to identify patients at high thrombotic risk, the SIC score, in contrast, particularly targets coagulation problems, the fundamental pathophysiological component of sepsis progression (22). However, because these scoring systems are essentially “intervalized” instruments based on weighted points, their capacity to precisely describe intricate, nonlinear interactions among continuous clinical variables is constrained. The external predictive efficacy of these ratings is further impacted by variations in treatment approaches and laboratory standards among sites. Furthermore, the majority of current scoring systems lack accurate, customized probability estimates for patient outcomes and are descriptive in nature. Therefore, incorporating multidimensional clinical factors and laboratory markers may provide complementary prognostic information for patients with SIC by enabling individualized risk estimation beyond conventional severity assessment frameworks.

Five predictors—coronary heart disease, serum potassium, PT-INR, platelet count, and platelet transfusion—were retained in the final multivariable model and incorporated into the nomogram. Rather than representing a single mechanistic pathway, these predictors capture complementary prognostic information across several clinically relevant pathophysiological domains. PT-INR and platelet count primarily reflect coagulation dysfunction and consumptive coagulopathy, serum potassium reflects disturbances in metabolic and renal homeostasis, coronary heart disease represents underlying cardiovascular reserve, and platelet transfusion serves as a treatment-related marker that may reflect the severity of thrombocytopenia, bleeding risk, and decompensated coagulopathy. By integrating these different dimensions within a multivariable logistic regression framework, the nomogram provides an individualized estimate of in-hospital mortality risk. Importantly, this multidimensional predictive integration should not be interpreted as evidence that these variables constitute a single biological pathway or have direct causal effects on mortality.

One important cardiovascular comorbidity that significantly increases risk in the pathophysiological course of SIC is coronary heart disease (CHD). Patients with CHD have compromised endothelial function and vulnerable microcirculation under conditions of systemic inflammation and hypercoagulability. These conditions make them more vulnerable to microthrombotic assaults, which exacerbates myocardial supply-demand mismatch and causes cardiac decompensation (23). The interaction between low cardiovascular reserve and SIC-induced inflammatory damage creates a positive feedback loop known as “coagulation disorder–circulatory failure–organ injury,” which not only directly jeopardizes survival but also exacerbates multiple organ dysfunction by impairing systemic perfusion (24). Clinically, CHD patients have a markedly increased risk of in-hospital death because to their decreased tolerance to volume resuscitation and hemodynamic support (25). Our analysis found that the non-survivor group had a much greater prevalence of CHD than the survivor group, which is consistent with these pathways and emphasizes the crucial role that decreased cardiovascular reserve plays in increasing the risk of SIC-associated mortality. Therefore, adding CHD to the predictive model improves the model's ability to provide a personalized prognostic assessment and makes it easier to identify high-risk patients with poor cardiovascular reserve.

Acid-base equilibrium, renal function, and cellular integrity are all integrated by serum potassium. Dysfunction of cellular membrane pumps causes extracellular potassium accumulation in the setting of microcirculatory impairment and tissue hypoxia brought on by SIC. This is made worse by decreased renal excretion, which puts patients at risk for hyperkalemia (26, 27). In addition to significantly raising the risk of fatal arrhythmias, this electrolyte imbalance puts patients at risk for sudden circulatory collapse when systemic inflammation is present (28). Potassium levels were considerably greater in non-survivors in the current study, which is not an isolated ionic fluctuation but rather an outward evidence of cellular metabolic collapse and multi-organ failure, which is a symptom of almost total breakdown of physiological homeostasis. Thus, adding serum potassium to the prediction model improves its capacity to detect rapid clinical deterioration and in-hospital mortality in SIC patients by capturing cross-system risk signals.

As a crucial clinical marker in the pathophysiology of SIC, PT-INR represents the extrinsic coagulation pathway's functional state and the liver's ability to produce clotting factors (29). The destabilization of hemostatic equilibrium is exacerbated by elevated PT-INR, which usually suggests higher consumption of coagulation components, reduced hepatic synthetic function, or anomalies in vitamin K-dependent pathways (30, 31). This change reflects the coagulation system's shift from a compensatory hypercoagulable state to a decompensated phase marked by concurrent factor depletion and synthetic malfunction, resulting in a “biphasic” disease where bleeding tendencies and microthrombosis coexist (32). PT-INR levels were considerably higher in non-survivors in the current investigation, indicating that this continuous variable captures important prognostic information related to coagulation dysfunction. In the multivariable model, the OR for PT-INR was expressed per 1.0-unit increase on its original scale; therefore, its relatively large numerical magnitude should be interpreted in the context of this substantial unit change. The wide confidence interval also indicates uncertainty in the magnitude of the effect estimate, and potential influences from sparse extreme values or a nonlinear relationship cannot be completely excluded. Incorporating PT-INR into the prediction model therefore contributes clinically relevant prognostic information regarding coagulation disturbance, although the magnitude of its individual effect estimate should be interpreted cautiously.

As a vital biological link between inflammatory reactions and thrombus formation, platelets are essential at the interface of immune control and the coagulation system (33). Thrombocytopenia in SIC patients is frequently caused by a variety of factors, such as splenic sequestration, immune-mediated destruction, bone marrow suppression, and consumptive coagulopathy (34). In addition to impairing basic hemostatic function, a decrease in platelet count indicates a high level of inflammation and excessive coagulation cascade activation. Furthermore, platelets, neutrophils, and endothelial cells can interact to create neutrophil extracellular traps, which can exacerbate endothelial damage and microvascular thrombosis in a vicious cycle (35). The body had reached a severe consumption-dominant decompensated phase in the current investigation, as evidenced by the significantly decreased platelet counts among non-survivors. Dysregulation of the immune–coagulation cross-talk is indicated by this simultaneous deficiency in platelet quantity and function, which exacerbates microcirculatory dysfunction. Thus, adding platelet count to the predictive model offers a crucial biological marker for assessing the probability of organ failure and systemic inflammatory damage in addition to enabling quantitative evaluation of hemostatic function.

In cases of severe thrombocytopenia or active bleeding, platelet transfusion is frequently used as a crucial clinical intervention. The choice to transfuse can be seen as an indirect measure of the severity of the illness and the state of decompensated coagulation rather than as evidence of a direct causal effect of platelet transfusion on mortality (36). In the present study, platelet transfusion was defined as platelet administration occurring within the first 24 h after SIC diagnosis, corresponding to the predefined predictor acquisition window. Therefore, this variable reflected information available during the initial risk stratification period rather than subsequent deterioration or terminal care. Frequent or large-scale platelet transfusions in SIC patients frequently indicate enduring systemic pathological processes, such as continuous microthrombus formation, coagulation factor consumption, and compromised hepatic synthetic function, indicating that the coagulation regulatory network has entered a phase of difficult-to-reverse imbalance (37). Previous research has shown that endothelial injury-mediated microcirculatory dysfunction and inflammation-driven hypercoagulable condition cannot be significantly improved by platelet supplementation alone. Consequently, organ perfusion and functional recovery may be restricted even with intensive transfusion treatment (38, 39). Furthermore, in critically sick patients, transfusion-related adverse responses, increased volume load, and possible immunomodulatory effects may worsen the respiratory and circulatory burden, so indirectly jeopardizing prognosis (40). According to these principles, a higher need for platelet transfusion is associated with an increased risk of in-hospital death and is more likely to reflect overall clinical deterioration and the advancement of multi-organ failure. In the current study, platelet transfusion was more frequent among non-survivors; however, this association should not be interpreted as evidence that platelet transfusion itself increases mortality. Transfusion demand may instead serve as a clinical surrogate marker of entry into a high-risk, decompensated disease phase. This association may also be influenced by confounding by indication, because patients with more severe coagulopathy are simultaneously more likely to receive platelet transfusion and to experience adverse outcomes. Furthermore, because platelet transfusion decisions were made during routine clinical care, differences in transfusion thresholds and practice patterns among participating centers may also have influenced this treatment-related predictor. This finding implies that exogenous supplementation alone is insufficient to stop systemic decompensation in the context of persistent inflammatory activation, impaired coagulation factor synthesis, and breakdown of tissue perfusion (41, 42). As a result, adding transfusion-related variables to predictive models makes it possible to capture dynamic risk signals throughout treatment, integrates clinicians’ thorough evaluation of disease severity and intervention intensity, and may contribute additional prognostic information for estimating the probability of in-hospital mortality and reflecting real-world clinical severity.

The current study has a number of advantages over earlier research on SIC prediction in terms of data source, methodological design, and clinical translatability. In order to reduce overfitting and improve robustness and generalizability, the model was first created using multicenter real-world clinical data that included a representative set of ICU patients with SIC. Its performance was then methodically assessed across training, testing, and external validation sets. Second, in order to ensure model parsimony while preserving predictive performance and biological interpretability, the study used a combined variable selection strategy using multivariable logistic regression and LASSO regression, closely adhering to the transparent reporting of a multivariable prediction model for individual prognosis or diagnosis (TRIPOD) guidelines. Additionally, the model incorporates important variables across several dimensions, such as coagulation function, organ function status, comorbidities, and the intensity of clinical interventions. This allows for the mapping of SIC's fundamental pathophysiological processes in addition to quantifying the risk of in-hospital mortality. The nomogram offers consistent net therapeutic benefit across pertinent choice thresholds, as further shown by the thorough model evaluation methodology, which includes ROC analysis, calibration curves, and decision curve analysis. Lastly, every predictor variable is regularly acquired from routine clinical and laboratory evaluations, allowing for quick bedside application and providing a useful decision-support tool for early risk assessment and resource distribution in the ICU.

There are a number of intrinsic limitations to this study. First, because this was a retrospective study, data collection was based on electronic medical records, which could contain missing information or inconsistent documentation. This could introduce selection and information biases and affect the accuracy of estimating associations between specific variables and outcomes. Additionally, patients who died within 24 h after ICU admission were excluded to minimize the influence of extremely early mortality on baseline assessment. However, this exclusion criterion may limit the generalizability of the model to patients with fulminant clinical deterioration or extremely rapid progression after ICU admission. Second, even though the model included multidimensional clinical and laboratory characteristics, patient prognosis may still be impacted by unmeasured or omitted potential confounders, such as genetic vulnerability, variations in treatment techniques, and variances in ICU care practices. Third, the model's usefulness for ongoing risk monitoring and disease progression assessment was limited because it was designed as a baseline prognostic model using static markers obtained within 24 h of SIC diagnosis. It therefore did not capture the longitudinal evolution of coagulation and organ function or patients’ time-dependent responses to subsequent therapies during the ICU stay. Future studies incorporating repeated measurements and dynamic treatment-response information may provide more comprehensive time-updated risk estimates. Furthermore, the study used a traditional logistic regression methodology rather than investigating deep learning or machine learning techniques, which could be useful for modeling intricate nonlinear interactions. Future studies should further evaluate whether integrating established severity scores, such as SOFA or APACHE II, with the proposed nomogram could provide additional incremental prognostic value. Lastly, the study population was mainly drawn from two hospitals in Hebei Province and one hospital in Chongqing City, despite the multicenter design; regional differences in the range of diseases, healthcare resources, and local treatment practices may impact the model's external applicability. Although the independent Chongqing cohort provided external validation across a different clinical setting, residual heterogeneity among institutions, including differences in patient characteristics, clinical management strategies, and transfusion practices, may still influence model transportability. In particular, center-specific platelet transfusion thresholds and routine transfusion practices could not be completely standardized in this retrospective study and may have introduced residual confounding related to the platelet transfusion predictor. Additional validation across larger populations and diverse healthcare systems is therefore necessary to further assess the generalizability of the model.

## 5 Conclusion

In this work, we developed and externally validated a nomogram for predicting in-hospital mortality in patients with SIC. The model demonstrated acceptable predictive performance and clinical utility across the development and validation cohorts. Based on the evaluation metrics, the nomogram showed reasonable discrimination and calibration and provides an individualized estimate of the probability of in-hospital mortality. As a complementary prognostic tool, the model integrates routinely available clinical information to support additional mortality risk stratification among patients with SIC. The visualization of multiple risk factors offers clinicians an intuitive approach for individualized prognostic assessment. As a result, it has great potential to improve prognosis by lowering mortality in this high-risk group and optimizing the use of healthcare resources.

## Data Availability

The data analyzed in this study is subject to the following licenses/restrictions: The data that support the findings of this study are available from the corresponding author upon reasonable request. Requests to access these datasets should be directed to Ruimin Tan, 1031829196@qq.com.

## References

[1] Singer M Deutschman CS Seymour CW Shankar-Hari M Annane D Bauer M. The third international consensus definitions for sepsis and septic shock (sepsis-3). JAMA. (2016) 315:801–10. 10.1001/jama.2016.0287 26903338 PMC4968574

[2] Yi Q Li YY Wu Y Li QY Luo SY Tang Y. Influence of coagulation dysfunction on prognosis of staphylococcus aureus sepsis in children. J Clin Pediatr. (2020) 38:44–8.

[3] Iba T Nisio MD Levy JH Kitamura N Thachil J. New criteria for sepsis-induced coagulopathy (SIC) following the revised sepsis definition: a retrospective analysis of a nationwide survey. BMJ Open. (2017) 7:e017046. 10.1136/bmjopen-2017-017046 28963294 PMC5623518

[4] Schmoch T Möhnle P Weigand MA Briegel J Bauer M Bloos F. The prevalence of sepsis-induced coagulopathy in patients with sepsis—a secondary analysis of two German multicenter randomized controlled trials. Ann Intensive Care. (2023) 13:3. 10.1186/s13613-022-01093-7 36635426 PMC9837358

[5] Parpucu UM Kucuk O Aydemir S. Factors influencing morbidity and mortality rates in tertiary intensive care units in Turkey: a retrospective cross-sectional study. Healthcare (Basel. (2024) 12:689. 10.3390/healthcare12060689 38540653 PMC10969906

[6] Tanaka C Tagami T Kudo S Takehara A Fukuda R Nakayama F. Validation of sepsis-induced coagulopathy score in critically ill patients with septic shock: *post hoc* analysis of a nationwide multicenter observational study in Japan. Int J Hematol. (2021) 114:164–71. 10.1007/s12185-021-03152-4 33895968 PMC8067778

[7] Zhou X Xie JF Yang Y. Mechanism and treatment progress for sepsis-induced coagulopathy. Chin J Intern Med. (2022) 61:224–8. 10.3760/cma.j.cn112138-20210314-00212 35090261

[8] Levi M Opal SM. Coagulation abnormalities in critically ill patients. Crit Care. (2006) 10:222. 10.1186/cc4975 16879728 PMC1750988

[9] Mumtaz H Ejaz MK Tayyab M Vohra LI Sapkota S Hasan M. APACHE scoring as an indicator of mortality rate in ICU patients: a cohort study. Ann Med Surg (Lond. (2023) 85:416–21. 10.1097/MS9.0000000000000264 37008173 PMC10060092

[10] Zhao H Dong Y Wang S Shen J Song Z Xue M. Comparison between sepsis-induced coagulopathy and sepsis-associated coagulopathy criteria in identifying sepsis-associated disseminated intravascular coagulation. World J Emerg Med. (2024) 15:190–6. 10.5847/wjem.j.1920-8642.2024.041 38855376 PMC11153374

[11] Ran X Zhang J Wu Y Du Y Bao D Pei H. Prognostic gene landscapes and therapeutic insights in sepsis-induced coagulopathy. Thromb Res. (2024) 237:1–13. 10.1016/j.thromres.2024.03.011 38513536

[12] Park SY. Nomogram: an analogue tool to deliver digital knowledge. J Thorac Cardiovasc Surg. (2018) 155:1793. 10.1016/j.jtcvs.2017.12.107 29370910

[13] Tan R Zhou Y Zhang S Yang J Du Q Wang J. Development and validation of a nomogram for early prediction of sepsis-induced coagulopathy: a multicenter study. Front Med (Lausanne. (2025) 12:1653699. 10.3389/fmed.2025.1653699 40995081 PMC12454385

[14] Iba T Arakawa M Levy JH Yamakawa K Koami H Hifumi T. Sepsis-induced coagulopathy and Japanese association for acute medicine DIC in coagulopathic patients with decreased antithrombin and treated by antithrombin. Clin Appl Thromb Hemost. (2018) 24:1020–6. 10.1177/1076029618770273 29695178 PMC6714750

[15] Jose RJ Williams AE Mercer PF Sulikowski MG Brown JS Chambers RC. Regulation of neutrophilic inflammation by proteinase-activated receptor 1 during bacterial pulmonary infection. J Immunol. (2015) 194:6024–34. 10.4049/jimmunol.1500124 25948816 PMC4456635

[16] Giustozzi M Ehrlinder H Bongiovanni D Borovac JA Guerreiro RA Gąsecka A. Coagulopathy and sepsis: pathophysiology, clinical manifestations and treatment. Blood Rev. (2021) 50:100864. 10.1016/j.blre.2021.100864 34217531

[17] Iba T Levi M Levy JH. Intracellular communication and immunothrombosis in sepsis. J Thromb Haemost. (2022) 20:2475–84. 10.1111/jth.15852 35979601 PMC9804233

[18] Kwaan HC. Microvascular thrombosis: a serious and deadly pathologic process in multiple diseases. Semin Thromb Hemost. (2011) 37:961–78. 10.1055/s-0031-1297375 22198861

[19] Patel P Walborn A Rondina M Fareed J Hoppensteadt D. Markers of inflammation and infection in sepsis and disseminated intravascular coagulation. Clin Appl Thromb Hemost. (2019) 25:1076029619843338. 10.1177/1076029619843338 30991817 PMC6714897

[20] Garcia-Alvarez M Marik P Bellomo R. Sepsis-associated hyperlactatemia. Crit Care. (2014) 18:503. 10.1186/s13054-014-0503-3 25394679 PMC4421917

[21] Iba T Arakawa M Mochizuki K Nishida O Wada H Levy JH. Usefulness of measuring changes in SOFA score for the prediction of 28-day mortality in patients with sepsis-associated disseminated intravascular coagulation. Clin Appl Thromb Hemost. (2019) 25:1076029618824044. 10.1177/1076029618824044 30808223 PMC6714949

[22] Iba T Umemura Y Wada H Levy JH. Roles of coagulation abnormalities and microthrombosis in sepsis: pathophysiology, diagnosis, and treatment. Arch Med Res. (2021) 52:788–97. 10.1016/j.arcmed.2021.07.003 34344558

[23] Volpe M Battistoni A Gabutti G Bonanni P Nati G Siliquini R. Cardiovascular disease related to influenza infection and the protective role of influenza vaccination. Int J Cardiol. (2026) 448:134178. 10.1016/j.ijcard.2026.134178 41539381

[24] Liu Y Yu M Shou S Chai Y. Sepsis-induced cardiomyopathy: mechanisms and treatments. Front Immunol. (2017) 8:1021. 10.3389/fimmu.2017.01021 28970829 PMC5609588

[25] Maimaitituersun G Abulimiti F Shao Q Zhou Y Jureti S Liu F. Machine learning and SHAP values for predicting coronary artery disease risk in xinjiang, China. Eur J Med Res. (2026) 31:281. 10.1186/s40001-025-03763-1 41545835 PMC12896198

[26] Levi M Toh CH Thachil J Watson HG. Guidelines for the diagnosis and management of disseminated intravascular coagulation. British committee for standards in haematology. Br J Haematol. (2009) 145:24–33. 10.1111/j.1365-2141.2009.07600.x 19222477

[27] Hoste EAJ Bagshaw SM Bellomo R Cely CM Colman R Cruz DN. Epidemiology of acute kidney injury in critically ill patients: the multinational AKI-EPI study. Intensive Care Med. (2015) 41:1411–23. 10.1007/s00134-015-3934-7 26162677

[28] Yamoldinov NR Dudarev MV Sarksyan DS Maleev VV. Cardiac arrhythmias in people who have had a coronavirus infection COVID-19: a review. Ter Arkh. (2023) 95:991–5. 10.26442/00403660.2023.11.202480 38158958

[29] Li C Huang W Han X Li H Qin X. The association between coagulation function and prognosis in patients with sepsis: a meta-analysis of predictive performance introduction. Front Med (Lausanne. (2025) 12:1706082. 10.3389/fmed.2025.1706082 41488071 PMC12756150

[30] Wada H Usui M Sakuragawa N. Hemostatic abnormalities and liver diseases. Semin Thromb Hemost. (2008) 34:772–8. 10.1055/s-0029-1145259 19214915

[31] Haran MZ Lichman I Berebbi A Weinmann E Rosenberg N. Unbalanced protein S deficiency due to warfarin treatment as a possible cause for thrombosis. Br J Haematol. (2007) 139:310–1. 10.1111/j.1365-2141.2007.06784.x 17897308

[32] Iba T Maier CL Scarlatescu E Levy JH. Introducing the new definition and diagnostic criteria of disseminated intravascular coagulation released by the international society on thrombosis and haemostasis in 2025. Semin Thromb Hemost. (2026) 52:10–7. 10.1055/a-2675-6068 40829630

[33] He Y Zhu W Xu T Liao Z Su J. Identification and immune responses of thrombocytes in bacterial and viral infections in grass carp (ctenopharyngodon idella). Fish Shellfish Immunol. (2022) 123:314–23. 10.1016/j.fsi.2022.03.009 35306178

[34] Song MJ Seo W- Jang Y Park S Ko RE Suh GY. RBC transfusion practices in critically ill patients with sepsis. Crit Care Med. (2025) 53:e2596–606. 10.1097/CCM.0000000000006892 41090988

[35] Portier I Campbell RA Denorme F. Mechanisms of immunothrombosis in COVID-19. Curr Opin Hematol. (2021) 28:445–53. 10.1097/MOH.0000000000000666 34232139 PMC8490282

[36] Drews RE. Critical issues in hematology: anemia, thrombocytopenia, coagulopathy, and blood product transfusions in critically ill patients. Clin Chest Med. (2003) 24:607–22. 10.1016/S0272-5231(03)00100-X 14710693

[37] Hamada SR Garrigue D Nougue H Meyer A Boutonnet M Meaudre E. Impact of platelet transfusion on outcomes in trauma patients. Crit Care. (2022) 26:49. 10.1186/s13054-022-03928-y 35189930 PMC8862339

[38] Iba T Helms J Okada H Nagakari K Sato K Ferrer R. Damage-associated molecular patterns, immunothrombosis, and intravascular inflammation in sepsis: a narrative integrative review. Semin Thromb Hemost. (2025). 10.1055/a-2776-5999 41418999

[39] Wu M Yan Y Xie X Bai J Ma C Du X. Effect of endothelial responses on sepsis-associated organ dysfunction. Chin Med J (Engl. (2024) 137:2782–92. 10.1097/CM9.0000000000003342 39501810 PMC11649274

[40] Zersen K. Fluid therapy in the critically ill patient. Vet Clin North Am Small Anim Pract. (2026) 56:245–55. 10.1016/j.cvsm.2025.08.006 40987687

[41] Huang C-Y Wu S-C Lin T-S Kuo P-J Yang JC-S Hsu S-Y. Efficacy of the geriatric trauma outcome score (GTOS) in predicting mortality in trauma patients: a retrospective cross-sectional study. Diagnostics (Basel. (2024) 14:2735. 10.3390/diagnostics14232735 39682643 PMC11639987

[42] Jiang T Liu K Chen Z. The impact of perioperative red blood cell transfusion on the prognosis of colorectal cancer. Front Surg. (2022) 9:927787. 10.3389/fsurg.2022.927787 35937600 PMC9349360

