# Supplementary material for: A simple nomogram for predicting in-hospital mortality in patients with sepsis-induced coagulopathy in the ICU: a multicenter retrospective study
Source: Front Med (Lausanne). 2026 Oct 29;13:1884945. doi: 10.3389/fmed.2026.1884945 (PMC13754807; doi:10.3389/fmed.2026.1884945)
Supplement: Supplementary file 1 [file Supplementaryfile1.docx]

**Supplementary Table**

Table S1. Comparison of baseline characteristics between the training and testing sets

| Variables | Training set (n=336) | Testing set (n=144) | Z/χ² | *P* |
| --- | --- | --- | --- | --- |
| Demographic data |  |  |  |  |
| Male, n(%) | 208 (61.90) | 87 (60.42) | χ²=0.09 | 0.759 |
| Age, SD | 73.00 (61.75, 81.00) | 71.00 (62.75, 83.00) | Z=-0.17 | 0.865 |
| Underlying diseases, n(%) |  |  |  |  |
| Coronary atherosclerotic heart disease | 67 (19.94) | 35 (24.31) | χ²=1.15 | 0.284 |
| Hypertension | 145 (43.15) | 54 (37.50) | χ²=1.33 | 0.249 |
| Diabetes | 80 (23.81) | 42 (29.17) | χ²=1.53 | 0.217 |
| Chronic obstructive pulmonary disease | 39 (11.61) | 20 (13.89) | χ²=0.49 | 0.485 |
| Cerebrovascular disease | 109 (32.44) | 52 (36.11) | χ²=0.61 | 0.435 |
| Liver dysfunction | 146 (43.45) | 57 (39.58) | χ²=0.62 | 0.432 |
| Chronic kidney disease | 88 (26.19) | 44 (30.56) | χ²=0.96 | 0.326 |
| Malignancy | 36 (10.71) | 23 (15.97) | χ²=2.58 | 0.108 |
| History of surgery within 3 months | 158 (47.02) | 74 (51.39) | χ²=0.77 | 0.380 |
| Infection sites, n(%) |  |  |  |  |
| Pulmonary | 224 (66.67) | 95 (65.97) | χ²=0.02 | 0.883 |
| Abdominal | 143 (42.56) | 55 (38.19) | χ²=0.79 | 0.373 |
| Blood | 48 (14.29) | 18 (12.50) | χ²=0.27 | 0.603 |
| Urinary system | 50 (14.88) | 32 (22.22) | χ²=3.84 | 0.050 |
| Central nervous system | 6 (1.79) | 1 (0.69) | χ²=0.25 | 0.618 |
| Skin and soft tissue | 10 (2.98) | 2 (1.39) | χ²=0.49 | 0.483 |
| Other | 16 (4.76) | 7 (4.86) | χ²=0.00 | 0.963 |
| ICU disease severity scores, M (Q₁, Q₃) |  |  |  |  |
| APACHE score | 23.00 (18.00, 28.00) | 24.00 (19.00, 29.00) | Z=-1.31 | 0.189 |
| SOFA score | 9.00 (7.00, 11.00) | 9.00 (7.00, 11.00) | Z=-0.05 | 0.957 |
| Vital signs, M (Q₁, Q₃) |  |  |  |  |
| Temperature(℃) | 36.50 (36.00, 37.30) | 36.60 (36.00, 37.30) | Z=-0.73 | 0.462 |
| HR(times/min) | 101.00 (85.00, 116.00) | 97.00 (83.75, 110.00) | Z=-1.31 | 0.192 |
| SBP(mmHg) | 121.00 (105.00, 140.00) | 122.50 (109.75, 140.00) | Z=-0.68 | 0.499 |
| DBP(mmHg) | 66.00 (57.00, 76.00) | 67.00 (56.00, 77.25) | Z=-0.23 | 0.817 |
| MAP(mmHg) | 85.00 (75.00, 95.33) | 85.67 (76.58, 93.00) | Z=-0.15 | 0.884 |
| RR(times/min) | 20.50 (16.00, 28.00) | 21.00 (16.00, 27.25) | Z=-0.12 | 0.901 |
| Laboratory tests, M (Q₁, Q₃) |  |  |  |  |
| pH | 7.36 (7.29, 7.42) | 7.37 (7.29, 7.42) | Z=-0.49 | 0.626 |
| lactate(mmol/L) | 2.80 (1.77, 4.88) | 2.50 (1.70, 4.35) | Z=-1.12 | 0.265 |
| AB(mmol/L) | 20.20 (16.88, 24.02) | 19.85 (16.20, 24.55) | Z=-0.09 | 0.932 |
| OI | 207.24 (145.25, 286.27) | 197.49 (131.81, 296.26) | Z=-0.67 | 0.502 |
| TP(g/L) | 49.20 (43.20, 54.95) | 48.85 (42.85, 55.10) | Z=-0.11 | 0.909 |
| ALB(g/L)(μmol/L) | 27.30 (23.17, 31.02) | 26.10 (23.40, 30.50) | Z=-0.55 | 0.580 |
| TBIL(μmol/L) | 21.30 (13.30, 37.95) | 17.95 (12.10, 27.02) | Z=-1.83 | 0.067 |
| DBIL(μmol/L) | 9.70 (5.40, 21.47) | 9.00 (4.80, 13.85) | Z=-1.42 | 0.156 |
| ALT(U/L) | 32.45 (17.00, 80.35) | 26.00 (14.00, 60.00) | Z=-2.32 | 0.020 |
| AST(U/L) | 60.70 (31.88, 136.00) | 57.50 (28.75, 115.38) | Z=-0.78 | 0.433 |
| K(mmol/L) | 3.92 (3.53, 4.40) | 3.95 (3.60, 4.57) | Z=-0.56 | 0.574 |
| Na(mmol/L) | 141.00 (136.07, 145.00) | 139.00 (134.07, 145.00) | Z=-2.03 | 0.042 |
| Cl(mmol/L) | 106.00 (102.00, 111.82) | 104.00 (100.62, 110.00) | Z=-2.24 | 0.025 |
| Ca(mmol/L) | 1.96 (1.84, 2.13) | 1.93 (1.81, 2.18) | Z=-0.34 | 0.736 |
| PO_4_(mmol/L) | 1.06 (0.75, 1.44) | 1.15 (0.91, 1.63) | Z=-2.45 | 0.014 |
| Mg(mmol/L) | 0.81 (0.70, 0.92) | 0.81 (0.67, 0.91) | Z=-0.52 | 0.600 |
| BUN(mmol/L) | 12.62 (8.50, 20.58) | 14.12 (8.68, 22.35) | Z=-1.17 | 0.244 |
| Cr(μmol/L) | 125.25 (78.35, 195.12) | 126.85 (80.78, 208.75) | Z=-0.18 | 0.858 |
| PCT(ng/mL) | 17.18 (3.04, 57.40) | 16.91 (2.86, 63.88) | Z=-0.07 | 0.946 |
| PT(s) | 16.30 (14.20, 18.33) | 16.20 (14.40, 18.12) | Z=-0.31 | 0.755 |
| PT-INR | 1.45 (1.30, 1.61) | 1.46 (1.35, 1.61) | Z=-0.89 | 0.371 |
| APTT(s) | 37.30 (32.90, 45.15) | 36.65 (32.10, 43.00) | Z=-1.26 | 0.209 |
| FIB(g/L) | 5.06 (3.23, 11.90) | 5.36 (3.55, 11.73) | Z=-0.70 | 0.482 |
| TT(s) | 15.80 (5.71, 17.22) | 15.60 (6.06, 16.90) | Z=-0.00 | 0.997 |
| WBC(10^9^/L) | 11.44 (5.93, 17.80) | 11.56 (6.27, 16.80) | Z=-0.06 | 0.951 |
| NEU(10^9^/L) | 10.09 (5.05, 16.50) | 9.80 (5.69, 15.34) | Z=-0.10 | 0.922 |
| LYM(10^9^/L) | 0.50 (0.30, 0.80) | 0.60 (0.35, 0.83) | Z=-1.06 | 0.291 |
| MON(10^9^/L) | 0.32 (0.14, 0.52) | 0.32 (0.12, 0.57) | Z=-0.03 | 0.979 |
| Hb(g/L) | 105.00 (88.00, 125.00) | 99.50 (89.00, 119.25) | Z=-1.01 | 0.312 |
| RDW(fL) | 48.10 (45.00, 54.23) | 48.35 (44.88, 54.40) | Z=-0.04 | 0.965 |
| PLT(10^9^/L) | 93.00 (55.75, 126.00) | 91.00 (53.75, 127.00) | Z=-0.43 | 0.670 |
| PDW | 16.10 (13.20, 17.22) | 16.05 (13.50, 17.62) | Z=-0.61 | 0.544 |
| Plateletcrit | 0.11 (0.07, 0.14) | 0.10 (0.07, 0.14) | Z=-0.70 | 0.481 |
| P-LCR | 37.55 (30.87, 45.12) | 38.25 (31.90, 46.90) | Z=-0.94 | 0.348 |
| MPV(fL) | 11.70 (10.70, 12.80) | 11.80 (10.88, 12.83) | Z=-0.88 | 0.378 |
| Interventions, n(%) |  |  |  |  |
| Deep venous catheterization | 277 (82.44) | 118 (81.94) | χ²=0.02 | 0.896 |
| Anticoagulant drugs | 147 (43.75) | 62 (43.06) | χ²=0.02 | 0.888 |
| Mechanical ventilation | 295 (87.80) | 124 (86.11) | χ²=0.26 | 0.611 |
| Hormones | 247 (73.51) | 106 (73.61) | χ²=0.00 | 0.982 |
| Vasoactive drugs | 321 (95.54) | 135 (93.75) | χ²=0.68 | 0.411 |
| CRRT | 188 (55.95) | 82 (56.94) | χ²=0.04 | 0.841 |
| Platelet transfusion | 88 (26.19) | 43 (29.86) | χ²=0.68 | 0.408 |
| Red blood cell transfusion | 154 (45.83) | 77 (53.47) | χ²=2.36 | 0.125 |
| Plasma infusion | 185 (55.06) | 78 (54.17) | χ²=0.03 | 0.857 |
| Infusion of cold precipitated clotting factors | 47 (13.99) | 22 (15.28) | χ²=0.14 | 0.712 |
| Human Fibrinogen Infusion | 4 (1.19) | 4 (2.78) | χ²=0.73 | 0.392 |
| Infusion of human albumin | 252 (75.00) | 112 (77.78) | χ²=0.42 | 0.515 |
| Hospitalization Status |  |  |  |  |
| ICU Stay(Days) | 6.00 (3.00, 13.00) | 6.00 (3.00, 13.25) | Z=-0.01 | 0.995 |
| Length of hospital stay(Days) | 16.00 (8.75, 27.00) | 16.00 (8.00, 27.50) | Z=-0.10 | 0.924 |
| Survival ending, n(%) |  |  | χ²=1.23 | 0.268 |
| Survival | 198 (58.93) | 77 (53.47) |  |  |
| Death | 138 (41.07) | 67 (46.53) |  |  |
| Z: Mann-Whitney test, χ²: Chi-square test | | | | |
| M: Median, Q₁: 1st Quartile, Q₃: 3st Quartile | | | | |
| APACHE II: acute physiology and chronic health evaluation II; SOFA: sequential organ failure assessment; HR: heart rate; SBP: systolic blood pressure; DBP: diastolic blood pressure; MAP: mean arterial pressure; RR: respiratory rate; AB, actual bicarbonate; OI, oxygenation index; TP: total protein; ALB: albumin; TBIL: total bilirubin; DBIL: direct bilirubin; ALT: alanine aminotransferase; AST: aspartate aminotransferase; K: potassium; Na: sodium; Cl: chloride; Ca: calcium; PO_4_: phosphorus; Mg: magnesium; BUN: blood urea nitrogen; Cr: creatinine; PCT: procalcitonin; PT: prothrombin time; PT-INR: prothrombin time-international normalisation ratio; APTT: activated partial thromboplastin time; FIB: fibrinogen; TT: thrombin time, WBC: white blood cells; NEU: neutrophils; LYM: lymphocytes; MON: monocytes; Hb: hemoglobin; RDW: red blood cell distribution width; PLT: platelet; PDW: platelet distribution width; P-LCR: platelet large cell ratio; MPV: mean platelet volume; CRRT: continuous renal replacement therapy; ICU: intensive care unit. | | | | |

Table S2. Baseline characteristics of candidate predictors in the validation set

| Variables | Death group (n=102) | Survival group (n=127) | Z/χ² | *P* |
| --- | --- | --- | --- | --- |
| Coronary heart disease, n(%) | 20 (19.61) | 29 (22.83) | χ²=0.350 | 0.554 |
| Serum potassium, M (Q₁, Q₃) | 3.97 (3.48, 4.62) | 4.02 (3.62, 4.46) | Z=-0.449 | 0.654 |
| PT-INR, M (Q₁, Q₃) | 1.54 (1.41, 1.81) | 1.44 (1.33, 1.52) | Z=-4.046 | <0.001 |
| PLT, M (Q₁, Q₃) | 75.00 (52.50, 111.25) | 116.00 (87.50, 133.00) | Z=-5.268 | <0.001 |
| Platelet transfusion, n(%) | 55 (53.92) | 25 (19.69) | χ²=29.170 | <0.001 |
| PT-INR, prothrombin time-international normalisation ratio; PLT: platelet. | | | | |

Table S3. Bootstrap stability analysis of predictor selection in the final nomogram

| Predictor | Bootstrap selection frequency |
| --- | --- |
| Coronary heart disease | 72.8% |
| Serum potassium | 86.5% |
| PT-INR | 98.4% |
| PLT | 96.7% |
| Platelet transfusion | 91.2% |
| PT-INR, prothrombin time-international normalisation ratio; PLT: platelet. | |

Table S4. Calibration performance of the nomogram across different cohorts

| Cohort | Calibration-in-the-large (95% CI) | Calibration slope (95% CI) | Brier score (95% CI) |
| --- | --- | --- | --- |
| Training cohort | 0.02 (-0.08 to 0.12) | 1.03 (0.90 to 1.16) | 0.132 (0.112 to 0.152) |
| Testing cohort | -0.06 (-0.21 to 0.09) | 0.94 (0.75 to 1.13) | 0.148 (0.118 to 0.178) |
| Validation cohort | 0.11 (-0.09 to 0.31) | 0.86 (0.63 to 1.09) | 0.156 (0.126 to 0.186) |

**Supplementary Figure**

**Figure S1A. ROC curve of the nomogram for predicting in-hospital mortality in the testing set.** The nomogram achieved an AUC of 0.776 (95% CI: 0.696–0.856), indicating acceptable discriminative performance in the testing cohort.

**Abbreviations:** ROC, receiver operating characteristic; AUC, area under the curve; CI, confidence interval.

**Figure S1B. ROC curve of the nomogram for predicting in-hospital mortality in the external validation set.** The nomogram achieved an AUC of 0.756 (95% CI: 0.692–0.819), demonstrating that the model retained acceptable discriminative performance in the independent external validation cohort.

**Abbreviations:** ROC, receiver operating characteristic; AUC, area under the curve; CI, confidence interval.

**Figure S2A. Calibration plot of the nomogram for predicting in-hospital mortality in the testing set.** The diagonal ideal line represents perfect agreement between predicted and observed probabilities. The apparent line represents the calibration performance in the original testing data, whereas the bias-corrected line represents the calibration estimate after bootstrap correction. Closer agreement between the model curves and the ideal line indicates better calibration.

**Figure S2B. Calibration plot of the nomogram for predicting in-hospital mortality in the external validation set.** The diagonal ideal line represents perfect agreement between predicted and observed probabilities. The apparent line represents calibration in the original validation data, whereas the bias-corrected line represents the calibration estimate after bootstrap correction. Closer agreement between the model curves and the ideal line indicates better calibration.

**Figure S3A. DCA of the nomogram for predicting in-hospital mortality in the testing set.** The x-axis represents the threshold probability and the y-axis represents net benefit. The nomogram curve represents the clinical net benefit of applying the model across different threshold probabilities. The “None” and “All” lines represent the strategies of assuming that no patients or all patients, respectively, will experience the outcome. A model curve above both reference strategies indicates potential clinical net benefit within the corresponding threshold range.

**Abbreviation:** DCA, decision curve analysis.

**Figure S3B. DCA of the nomogram for predicting in-hospital mortality in the external validation set.** The x-axis represents the threshold probability and the y-axis represents net benefit. The nomogram curve represents the clinical net benefit of applying the model across different threshold probabilities. The “None” and “All” lines represent the strategies of assuming that no patients or all patients, respectively, will experience the outcome. A model curve above both reference strategies indicates potential clinical net benefit within the corresponding threshold range.

**Abbreviation:** DCA, decision curve analysis.
